# Supplementary material for: CBEC inventory optimization model design based on spatiotemporal attention and transformer architecture
Source: PLoS One. 2026 Feb 2;21(2):e0338951. doi: 10.1371/journal.pone.0338951 (PMC12863534; doi:10.1371/journal.pone.0338951)
Supplement: Attachment — Submitted filename: PONE-D-25-36724+response to reviewers.docx [file pone.0338951.s002.docx]

**Comments to the Author**

5. Review Comments to the Author

Please use the space provided to explain your answers to the questions above. You may also include additional comments for the author, including concerns about dual publication, research ethics, or publication ethics. (Please upload your review as an attachment if it exceeds 20,000 characters)

**Response to Reviewers**

**Manuscript ID: PONE-D-25-36724
Title: Cross-border e-commerce inventory optimization model design based on Spatiotemporal attention and Transformer Architecture
Authors: Zongping Lin, Yingyi Huang, Jing Yang, Chunhu Cui, Yabin Lian, Honglei Zhang**

**We sincerely thank the reviewers for their insightful comments and constructive suggestions, which have significantly improved the quality of our manuscript. We have carefully addressed each comment and revised the manuscript accordingly. Below is a point-by-point response to the reviewers’ comments.**

Reviewer #1: The manuscript presents a well-structured and innovative approach to cross-border e-commerce inventory prediction using a spatiotemporal perception Transformer model. The integration of spatial and temporal self-attention mechanisms combined with multi-scale diffusion convolution effectively addresses challenges in capturing complex spatiotemporal dependencies. The methodological rigor is demonstrated through comprehensive experiments, including comparisons with state-of-the-art models and detailed ablation studies, which validate the contributions of each model component. The use of robust evaluation metrics (MAE, MAPE, RMSE) and the choice of Huber Loss for handling outliers strengthen the reliability of the results. The presentation is clear, and the findings are well supported by the data. Minor improvements could include clearer explanations of some complex formulae for broader accessibility and a deeper discussion on potential limitations and future directions. Overall, the paper makes a significant contribution to the field of inventory forecasting in cross-border e-commerce and is suitable for publication with minor revisions.

Reply: We thank the reviewer for the positive feedback and valuable suggestions. We have made the following improvements to the manuscript:

**4.4  Limitations**

Although the proposed model demonstrates superior performance in cross-border e-commerce inventory prediction, several limitations remain to be addressed in future work.

First, the computational complexity of the self-attention mechanism remains a challenge, especially when dealing with large-scale spatial networks or long-term historical data. Although our model achieves a balance between performance and efficiency compared to other Transformer-based baselines, the quadratic complexity of self-attention with respect to sequence length may still hinder its application in real-time scenarios or on edge devices with limited computational resources. Future work will explore more efficient attention mechanisms, such as linear attention or sparse attention, to reduce computational overhead without significantly sacrificing accuracy.

Second, the current model primarily relies on historical inventory data and basic temporal features (e.g., time of day, day of week). While these features capture major trends, they do not fully incorporate external factors that may significantly impact inventory demand, such as sudden market fluctuations, promotional activities, macroeconomic policies, or unexpected events. In the future, we plan to enhance the model by integrating multi-source data, including social media trends, search engine indexes, and economic indicators, to improve the robustness and adaptability of predictions under volatile conditions.

Third, the model's performance is dependent on the quality and granularity of the data. In practice, cross-border inventory data often suffer from missing values, noise, and inconsistent recording frequencies across different regions or platforms. Although we used Huber Loss and interpolation to mitigate some of these issues, a more sophisticated data imputation or denoising module could be beneficial. Future research could explore joint modeling of data reconstruction and prediction within an end-to-end framework, or leverage generative model to synthesize realistic training data under sparse observations.

Lastly, the current model is designed and validated primarily for inventory prediction in the fashion and consumer goods sectors. Its effectiveness in other industries with different demand patterns remains to be verified. We intend to extend the evaluation to more diverse product categories and explore domain adaptation techniques to improve the generalizability of the model across different industrial contexts.

Reviewer #2: The topic fits PLOS ONE’s scope of methodologically sound research with potential practical relevance. This work could be suitable for publication – with improved transparency, statistical rigour, and language clarity.

**Reply: Thank you for your valuable feedback. We have made revisions to the reviewer's comments. I hope the revised version can be published.**

Recommendations:
1. Please specify exact train/validation/test periods (calendar dates) and whether a rolling-origin evaluation was used. State the prediction horizon TTT and the input window length; list all covariates and preprocessing (imputation, scaling).

Please specify exact train/validation/test periods (calendar dates) and whether a rolling-origin evaluation was used. State the prediction horizon TTT and the input window length; list all covariates and preprocessing (imputation, scaling).

**Reply: Thank you for your valuable feedback. We have added relevant data processing procedures in the revised manuscript.**

The experimental dataset spans from January 1, 2022, to December 31, 2023. We employed a fixed-origin split for evaluation, with the training set covering January 1, 2022, to September 30, 2023, the validation set from October 1, 2023, to November 30, 2023, and the test set comprising the final month, December 1 to December 31, 2023. The model's input window length was set to 28 days, meaning it uses the preceding 28 days of data to forecast a prediction horizon (T) of 7 days. The covariates used include historical inventory levels, order volume, production volume, and temporal features (day of the week and holiday indicators). For preprocessing, minimal missing values (<0.5%) were handled via linear interpolation, and all numerical features were normalized to the [0, 1] range using Min-Max scaling fitted solely on the training data to prevent leakage.

2. Please document the implementations used (citations/repos), hyper-parameter grids, and early-stopping criteria for each baseline. Ensure the same feature sets and horizons are used across models.

**Reply: Thank you for your valuable feedback. We have added relevant data processing procedures in the revised manuscript.**

To ensure a fair and consistent comparison, all baseline models and the proposed model were implemented using the same feature sets, input window length (28 days), and prediction horizon (7 days). The implementations of the baseline models were based on their official open-source repositories: GWNET^[18]^, STSGCN^[17]^, and T-GCN^[19]^ were sourced from their respective authors' GitHub pages. Hyperparameter tuning for all models was conducted via a grid search over key parameters: $\text{learning rate}\in\left\{0.001,\text{ }0.0005\right\}$, $\text{hidden units}\in\left\{32,\text{ }64\right\}$, and $\text{number of layers}\in\left\{2,\text{ }3\right\}$. Training was regulated by an early-stopping criterion with a patience of 10 epochs, monitoring the validation loss to avoid overfitting and ensure convergence.

3. Fix all equation typesetting and define every symbol on first use. If sinusoidal positional encoding deviates from the standard base, justify the choice. Remove or explain the reference to ILOG CPLEX (seems unrelated to the deep model training) to avoid confusion.

**Reply: We sincerely thank the reviewer for these meticulous and crucial suggestions. We have thoroughly revised the manuscript to address these points, which have significantly improved the clarity and rigor of our work. Please find our point-by-point responses below:**

**3.1 Fix all equation typesetting and define every symbol on first use.**

Where $A\in\text{R}^{N\times N}$ is the adjacency matrix, $I_{N}$ is the identity matrix, and the degree matrix $D\in\text{R}^{N\times N}$ is a diagonal matrix composed of node degrees. The eigenvalue composition of matrix $\Lambda$ for matrix $L$ is a diagonal matrix, and $U$ is the Fourier basis. Here, linear projections on the minimum $k$non trivial feature vectors are used to generate spatial Turalaplus embeddings, which are then extended to the input data dimension through fully connected layers, denoted as $X_{S}\in\text{R}^{T\times N\times d}$. Afterwards, the spatial information is encoded using the position encoding method in the traditional Transformer model, as shown:

**3.2 Remove or explain the reference to ILOG CPLEX (seems unrelated to the deep model training) to avoid confusion.**

On a laptop computer with an Intel (R) Core (TM) i7-10510U processor, a clock speed of 1.8 GHz, and 16 GB of memory, all model training and inference experiments were conducted using the PyTorch deep learning framework within the Jupyter Notebook 6.4.6 environment.
4. Please standardise terminology (e.g., “cross-border e-commerce (CBEC)”), expand acronyms at first mention, and check reference formatting.

**Reply: Thank you for your valuable feedback. We have conducted a comprehensive review of the manuscript.**

**SASA:** Spatial Aware Self-attention

**TASA:** Temporal Aware Self-attention

**MDC:** Multi-scale Diffusion Convolution

**ARIMA:** Autoregressive Integrated Moving Average

**GCN:** Graph Convolutional Network
